# Retraction: Yiqi Fumai lyophilized injection attenuates doxorubicin-induced cardiotoxicity, hepatotoxicity and nephrotoxicity in rats by inhibition of oxidative stress, inflammation and apoptosis

**DOI:** 10.1039/d5ra90003d

**Published:** 2025-01-13

**Authors:** Yue Gu, Aichun Ju, Bingjie Jiang, Jingze Zhang, Shuli Man, Changxiao Liu, Wenyuan Gao

**Affiliations:** a Tianjin Key Laboratory for Modern Drug Delivery and High-Efficiency, School of Pharmaceutical Science and Technology, Tianjin University Weijin Road Tianjin 300072 China pharmgao@tju.edu.cn +86-22-87401895 +86-22-87401895; b Tasly Pride Pharmaceutical Company Limited Tianjin 300410 China; c Department of Pharmacy, Logistics University of Chinese People’s Armed Police Forces Tianjin 300309 China zhangjingze1877@163.com +86-22-84876773; d State Key Laboratory of Food Nutrition and Safety, College of Biotechnology, Tianjin University of Science & Technology Tianjin China msl@tust.edu.cn +86-22-60601265; e The State Key Laboratories of Pharmacodynamics and Pharmacokinetics Tianjin 300193 China

## Abstract

Retraction of ‘Yiqi Fumai lyophilized injection attenuates doxorubicin-induced cardiotoxicity, hepatotoxicity and nephrotoxicity in rats by inhibition of oxidative stress, inflammation and apoptosis’ by Yue Gu *et al.*, *RSC Adv.*, 2018, **8**, 40894–40911, https://doi.org/10.1039/C8RA07163B.

The Royal Society of Chemistry, with the agreement of the authors below, hereby wholly retracts this *RSC Advances* article due to concerns with the reliability of the data.

There are several duplications of image panels within this paper with images published in other papers by different authors; Fig. 7C YQFM/TGF-β1 panel with ref. 1, Fig 11C YQFM/Liver with ref. 2, Fig 11B Control/Liver with ref. 3, Fig 11C DOX/Liver with ref. 3, Fig 11B YQFM/Heart with ref. 4 and Fig 11F western blot GADPH band with ref. 5.

Within the paper, there are many duplications including the Fig. 10A DOX/Kidney (COX-2) panel with the Fig. 11D DOX/Kidney (Cytochrome C) panel, the Fig. 11A DOX + YQFM/Heart (BAX) panel with the Fig. 11B DOX + YQFM/Heart (BCl-2) panel, and the BCl-2 western blot bands within Fig. 11F and G.

The authors state that they obtained the images in Fig. 7C, 8A–L, 10A, 11A–G and 12A–L from a third party.

Given the significance of these concerns, the Editor has lost confidence that the findings presented in this paper are reliable.

Signed: Yue Gu, Bingjie Jiang, Shuli Man, Aichun Ju, Jingze Zhang, Wenyuan Gao

Date: 17th December 2024

Retraction endorsed by Laura Fisher, Executive Editor, *RSC Advances*
